# Emergence of coexisting *bla*_NDM_ and *mcr-1* genes in *Escherichia coli* isolates from the guts of healthy individuals

**DOI:** 10.1128/spectrum.02014-25

**Published:** 2025-12-23

**Authors:** Shuang Wang, Lu Liu, Lei Du, Shanli Gao, Xiaolin Yu, Lixiao Cheng, Yuzhen Chen, Zengqiang Kou, Wenkui Sun

**Affiliations:** 1 Infection Disease Control of Institute, Shandong Center for Disease Control and Prevention 373247, Jinan, China; 2 Division of Liver Diseases, Shandong Public Health Clinical Center, Jinan, China; 3 Central Office, Zhucheng Center for Disease Control and Prevention, Weifang, China; Yan'an University, Yan'an, Shaanxi, China

**Keywords:** drug resistance, NDM, *mcr-1*, *Escherichia coli*, healthy individuals

## Abstract

The emergence of *bla*_NDM_/*mcr-1*-positive *Escherichia coli* poses a huge challenge to infection control. The purpose of this study was to investigate the plasmid and genomic characteristics of *bla*_NDM_/*mcr-1*-positive *E. coli* isolates from the gut of healthy individuals. This study performed drug susceptibility testing, whole-genome sequencing (WGS), analysis of resistance genes, characterization of plasmids, and single-nucleotide polymorphism (SNP) analysis on *bla*_NDM_/*mcr-1*-positive *E. coli* isolates collected from the gut of 628 healthy individuals. Our results showed that all seven isolates were extensively drug-resistant, with susceptibility only to tigecycline and streptomycin. Notably, WGS revealed that *bla*_NDM_ and *mcr-1* were located on transferable plasmids, and SNP analysis placed the isolates in five distinct clades. These findings underscore the need for surveillance to monitor dissemination among healthy populations.

**IMPORTANCE:**

This study reports isolation of *Escherichia coli* strains coharboring the critical resistance genes *bla*_NDM_ (carbapenemase) and *mcr-1* (colistin resistance) from the guts of healthy individuals (rural China). All seven isolates were extensively drug resistant, susceptible only to tigecycline and streptomycin. Crucially, both resistance genes were located on transferable plasmids, demonstrating potential for horizontal spread. Genomic analysis revealed diverse strains and plasmid types carrying these genes within mobile genetic contexts. This discovery identifies healthy human carriers as a significant reservoir for pan-resistant bacteria, posing severe public health challenges and necessitating urgent surveillance in endemic areas.

## OBSERVATION

The further spread of carbapenemase/*mcr-1*-positive Enterobacteriaceae could lead to a rise in untreatable infections caused by gram-negative bacteria (1). The healthy human gut commensal microbiota has been described as a reservoir of antibiotic-resistance genes (ARGs) (2). In this study, we discovered the coexistence of *bla*_NDM_ and *mcr-1* genes in *Escherichia coli* isolates from healthy individuals. We performed an in-depth genomic analysis to explore the genetic contexts and molecular characteristics of the plasmids harboring *bla*_NDM_ and *mcr-1*. Notably, the *bla*_NDM_ and *mcr-1* genes located on the plasmids could be transferred from each of the isolates to recipient strains via conjugation. To our knowledge, this is the first report of *bla*_NDM_/*mcr-1*-positive *E. coli* from the guts of healthy individuals in rural communities. The patterns of antibiotic resistance and transmission in *bla*_NDM_/*mcr-1*-positive *E. coli*, particularly in healthy individuals, should be closely monitored.

In 2023, we sampled 628 non-duplicate stool samples from 628 healthy subjects from a rural community in Shandong Province and screened for carbapenemase-positive Enterobacteriaceae isolates. The demographic characteristics and geographic information of the surveyed participants are presented in Tables S1 and S2. Isolate identification was performed using matrix-assisted laser desorption ionization time-of-flight mass spectrometry. The carbapenemase-encoding gene (*bla*_NDM_) and *mcr-1* gene were identified using PCR (primer sequences for *bla*_NDM_: forward, ATGGAATTGCCCAATATTATGCAC; reverse, TCAGCGCAGCTTGTCGGC; primer sequences for *mcr-1*: forward, GGTGGCGTTCAGCAGTCA; reverse, GCAGATGGCGTTGTTGGT). The PCR products were purified using a PCR Purification Kit (Qiagen) and Sanger sequenced to confirm genetic identity. The minimum inhibitory concentrations of the *E. coli* isolates for meropenem, colistin, ertapenem, ceftazidime-avibactam, tigecycline, cefotaxime, ceftazidime, ciprofloxacin, azithromycin, chloramphenicol, nalidixic acid, streptomycin, trimethoprim-sulfamethoxazole, amikacin, ampicillin, ampicillin-sulbactam, and tetracycline were determined using the broth microdilution method according to the European Committee on Antimicrobial Susceptibility Testing standards (version 12.0, 2022). *E. coli* ATCC 25922 was used as the quality control strain.

Whole-genome sequencing (WGS) was performed using Illumina NovaSeq 6000 and PacBio platforms at Novogene (Beijing, China). Hybrid assembly of Illumina and PacBio reads was performed using CLC Assembly Cell software (v3.2.2) and SMRT Link (v.5.0.1). Resistance genes were identified *in silico* using the Antibiotic Resistance Genes Database and ResFinder (3). Insertion sequence (IS) elements and integrons were identified using ISfinder (https://www-is.biotoul.fr/) and MobileElementFinder (https://cge.food.dtu.dk/services/MobileElementFinder/). BacWGSTdb provided online analyses of multilocus sequence typing (4). To obtain sequence information for the plasmids, comparisons with the National Center for Biotechnology Information (NCBI) nucleotide database (NT) were performed using the BLAST tool on the NCBI website (http://blast.ncbi.nlm.nih.gov/Blast.cgi?PROGRAM=blastn&PAGE_TYPE=BlastSearch&LINK_LOC=blasthome). Plasmid and chromosome sequences were differentiated by sequence length and alignment, with plasmid circularization confirmed by resequencing depth. Plasmid size was determined by calculating the number of bases in the plasmid sequence. Comparative analysis of different plasmids harboring the *bla*_NDM_/*mcr-1* genes was performed using the Blast Ring Image Generator. The kSNP3 analysis software (with the kmer_length parameter set to 13) was used for single-nucleotide polymorphism (SNP) analysis and phylogenetic tree construction. The phylogenetic tree was then refined and visualized using Evolview (5). The transfer of two resistance genes (*bla*_NDM_ and *mcr-1*) was studied using standard conjugation for all seven isolates.

**Fig 1:**
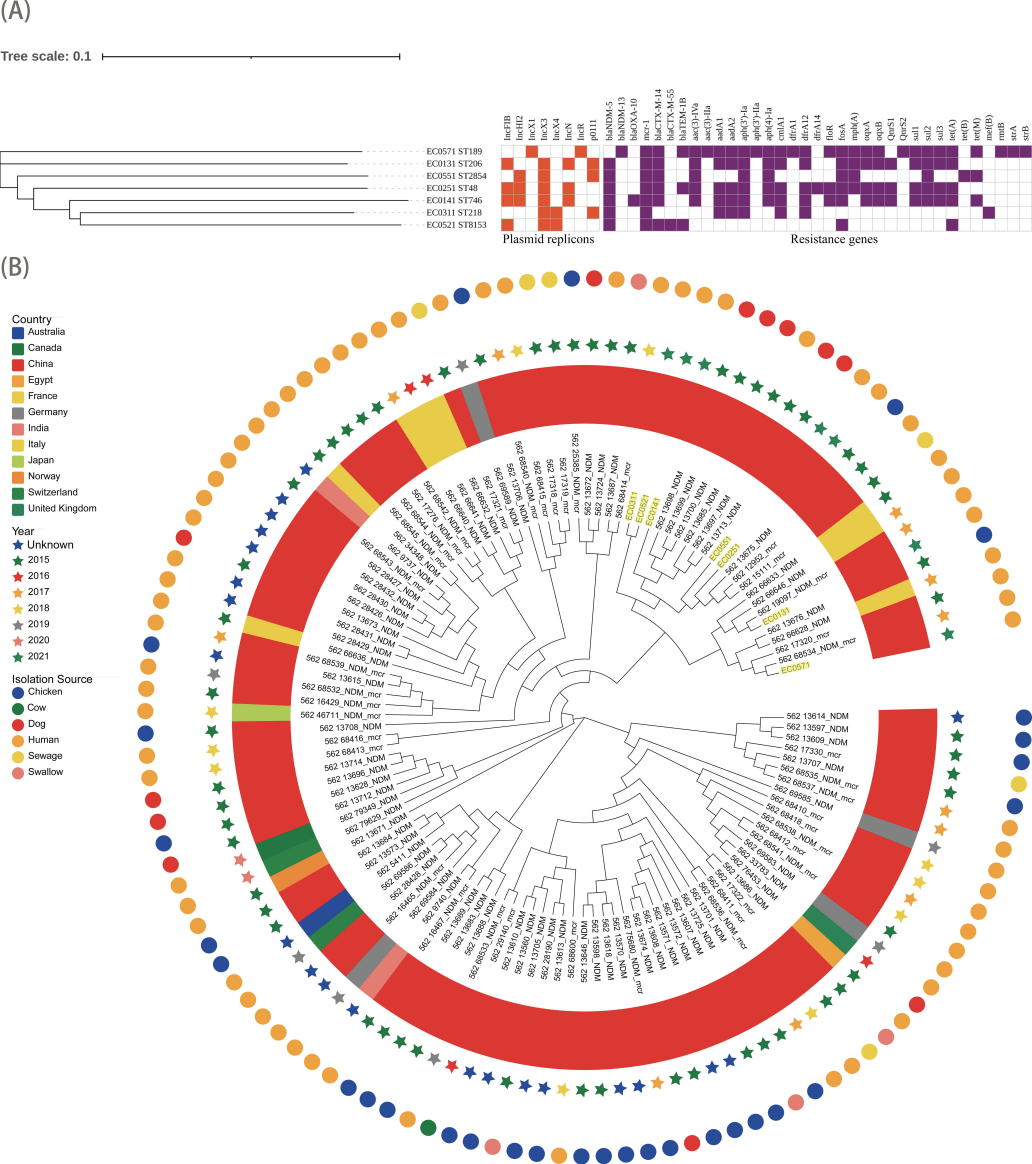
(**A**) ARGs and types of plasmids among *bla*_NDM_/*mcr-1*-positive *E. coli* isolates across the phylogenetic tree. (**B**) Phylogenetic tree of 7 *bla*_NDM_/*mcr-1*-positive *E. coli* isolates in this study and another 113 *bla*_NDM_-positive *E. coli* isolates in the NCBI database. Abbreviations: ARG, antibiotic resistance gene; NCBI, National Center for Biotechnology Information.

Seven *bla*_NDM_/*mcr*-1-positive *E. coli* isolates were obtained from seven healthy subjects across 12 villages, originating from six different villages. All seven isolates exhibited resistance to ertapenem, meropenem, colistin, cefotaxime, ceftazidime, ceftazidime-avibactam, tetracycline, azithromycin, ampicillin, and ampicillin-sulbactam. However, all isolates remained susceptible to tigecycline and streptomycin. Antimicrobial susceptibility testing revealed that all isolates were extensively drug resistant, defined as non-susceptibility to at least one agent in all but two or fewer antimicrobial classes (6). The results of antimicrobial susceptibility testing are shown in Table S3. WGS results showed that the seven *bla*_NDM_/*mcr*-1-positive *E. coli* isolates harbored multiple ARGs (Fig. 1B). The most prevalent carbapenemase gene detected was *bla*_NDM-5_ (*n* = 6), followed by *bla*_NDM-13_ (*n* = 1) and *bla*_OXA-10_ (*n* = 1). Notably, one isolate harbored both *bla*_NDM-5_ and *bla*_OXA-10_. Three ESBL genes were detected: *bla*_CTX-M-14_ was identified in six isolates, *bla*_CTX-M-55_ in one isolate, and *bla*_TEM-1B_ in two isolates. Plasmid-mediated quinolone resistance genes (*oqxA*, *oqxB*, *qnrS1*, and *qnrS2*) were detected in four isolates. Sulfonamide resistance genes *sul1*, *sul2*, and *sul3* co-occurred in four isolates. Six isolates carried multiple aminoglycoside resistance genes, with *aph(3′)-Ia* being the most common. Furthermore, the *bla*_NDM_ and *mcr-1* genes were transferable from each isolate into recipient *E. coli* strains via conjugation, confirmed by PCR. The transfer frequency was 4.5 × 10^−8^ for *bla*_NDM_-positive plasmids and 3.6 × 10^−8^ for *mcr-1*-positive plasmids.

The genetic similarities between the 7 isolates in this study and another 113 *bla*_NDM_-positive *E. coli* isolates from various sources (human, chicken, dog, swallow, and cow) in previous studies available in public databases were characterized. SNP analysis showed that the *bla*_NDM_/*mcr-1*-positive *E. coli* isolates in this study clustered into five lineages within a major clade, with the minimum pairwise difference (between EC0131 and EC0521) being 341 SNPs. The ST206 *E. coli* isolate EC0131 clustered with clinical isolates of human origin from Zhejiang Province, China, showing a difference of 1,365 SNPs. The isolate EC0571 differed by 1,448 SNPs from a *bla*_NDM_/*mcr-1* positive *E. coli* isolate from a patient in Hong Kong SAR, China. The remaining 111 isolates were more distantly related to the isolates in this study, with differences ranging between 2,176 and 37,675 SNPs (Fig. 1B).

**Fig 2:**
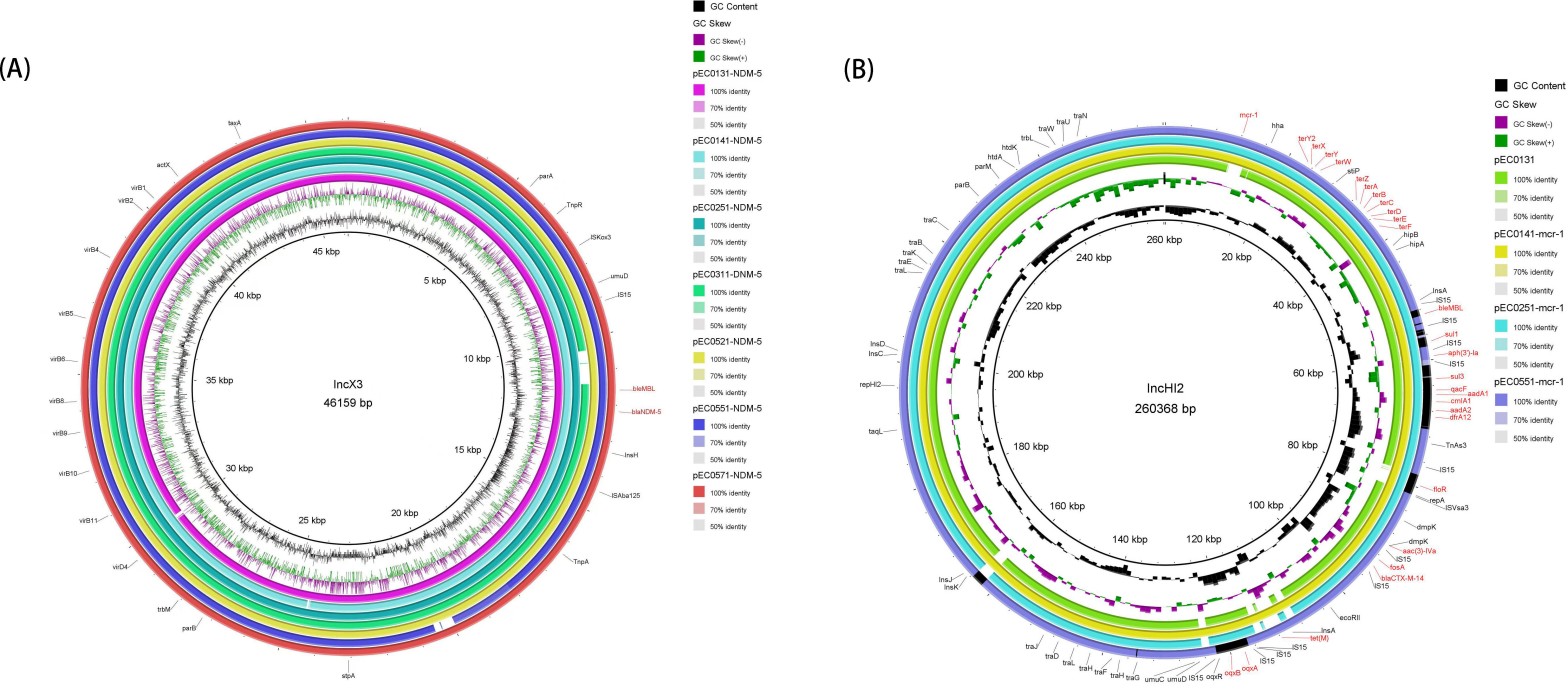
Circular alignments of homologous contigs from *bla*_NDM_/*mcr*-1-positive plasmids in this study. (**A**) IncX3 plasmid. (**B**) IncHI2 plasmid (contains *mcr*-1-negative plasmid pEC0131). Note: Each ring represents the corresponding plasmids shown in each figure, and plasmid types are given in the center of the rings.

In this study, the presence and genetic locations of *bla*_NDM_/*mcr-1* in seven isolates were determined through hybrid Illumina and PacBio platform sequencing data analysis. All isolates harbored the carbapenem resistance gene *bla*_NDM_ on their plasmids. The *bla*_NDM_-positive plasmids were all IncX3 type, ranging from 45 to 126 kb in size, and exhibited remarkably high sequence similarity (99.80%– 100.0% pairwise nucleotide sequence identity) (Fig. 2A). Of the *mcr-1* genes in the seven isolates, three were located on IncHI2-type plasmids; one was located on an IncX4-type plasmid; and the other three were located on chromosomes. Two plasmids (pEC0141-*mcr-1* and pEC0251-*mcr-1*) harboring *mcr-1* genes displayed high sequence similarity (96% sequence coverage and 100% nucleotide identity). They shared the same genetic arrangement: *dfrA12-*IS*15-floR-aac (3)-IVa-*IS*15-fosA-bla*_CTX_*-*_M-14_*-*IS*15-ecoRII-*IS*15-oqxA/B* (Fig. 2B).

The insertion sequences IS*Kox3*, IS*15*, and IS*Aba125* were located both upstream and downstream of *bla*_NDM-5_ within the IncX3 plasmids. As a characteristic element surrounding *bla*_NDM_, IS*Aba125* typically formed a composite transposon named Tn*125*, with the *bla*_NDM_ type IV secretion system genes cluster (*virB1*/ *virB2*/ *virB4*/ *virB5*/ *virB6*/ *virB8*/ *virB9*/ *virB10*/ *virB11*/ *virD4*) located upstream of the *bla*_NDM-5_ gene (7). The genetic arrangement of plasmids harboring *bla*_NDM-5_ genes was consistent: *parA*-IS*Kox3-umuD*-IS*15-ble*_MBL_*-bla*_NDM-5_*-insH*-IS*Aba125-tnpA-stpA-parB-trbM-virD4virB11-virB10-virB9-virB8-virB6-virB5-virB4-virB2-virB1-taxA*. These plasmids were similar to the *bla*_NDM-5_-carrying IncX3 plasmid pKP-13-8-NDM-5 (99.80%–99.99% nucleotide sequence identity and 90%–100% coverage) from a *Klebsiella pneumoniae* isolate from a patient blood sample in Henan Province, China, in 2020 (GenBank: MN175389.1). Additionally, genetic environment characterization revealed that *bla*_NDM-5_ in the plasmid pEC0131-NDM-5 was located in a class 1 integron named In*907*, with a conserved module structure of IS*15-ble*_MBL_-*bla*_NDM-5_*-CH*-IS*Aba125*. Studies have reported that this conserved structure may be strongly correlated with the horizontal transfer of *bla*_NDM_-carrying plasmids (8). Three plasmids carrying *mcr-1* harbored tellurium resistance-encoding genes (*terZ*, *terA*, *terB*, *terC*, *terD*, *terE*, *terW*, *terY*, *terX*, and *terF*), which reportedly provide protection against the toxic effects of tellurite, enabling bacteria to survive in tellurite-rich environments (9). Aminoglycoside resistance genes [*aac(3)-IV* and *aph(3')-Ia*], beta-lactam resistance gene (*bla*_CTX_*_-_*_M-14_), sulfonamide resistance gene (*sul*), and colistin resistance gene (*mcr-1*) were located in these multidrug-resistant plasmids. Three clusters of *tra*-encoding genes (*traG/H/F/L/D/J*, *traL/E/K/B/C*, and *traW/U/N*) might provide the conjugation machinery to the bacterial cell and are involved in the translocation of proteins such as virulence factors and other elements (10). We obtained seven *bla*_NDM_/*mcr-1*-positive *E. coli* isolates with different STs. The *bla*_NDM_ and *mcr-1* genes in several isolates were located within the same genetic environment on identical plasmid types. This phenomenon is likely driven by horizontal gene transfer of conjugative plasmids between strains. In the seven isolates tested, *bla*_NDM_ and *mcr-1* were located on separate IncX3 and IncHI2 plasmids, respectively. This finding suggests that plasmids carrying *bla*_NDM_ and *mcr-1* can mobilize and disseminate ARGs via multiple mobile genetic elements (such as transposons and insertion sequences) under strong antibiotic selective pressure.

Our study has several limitations. First, the number of strain genomes downloaded from the database was limited, so no strains closely related to the genetic relationship of the studied isolates were acquired. Second, delays in isolate analysis may have affected the accuracy of temporal trends in resistance gene dissemination. Third, the unavailability of clinical metadata, including antibiotic usage history, underlying health conditions, or travel history, constrained our ability to identify potential risk factors associated with carriage. Finally, the lack of animal or environmental sampling prevents assessment of potential zoonotic or environmental reservoirs, which is important for understanding the transmission potential of resistance genes in animal hosts and environmental reservoirs.

This study reveals the prevalence and transmission potential of *bla*_NDM_/*mcr-1*-positive *E. coli* among healthy individuals in a rural community in China. The coexistence of *bla*_NDM_ and *mcr-1* in Enterobacterales poses a critical public health threat, as their rapid dissemination to clinical strains could compromise the efficacy of last-resort antibiotics. Our findings emphasize the need for a One Health approach to surveillance, integrating human, animal, and environmental monitoring to curb the spread of resistance. Public health interventions, such as antibiotic stewardship programs and community education, are urgently required in endemic regions. Future studies should explore zoonotic and environmental transmission routes to inform targeted control measures.

## Data Availability

All draft genomes were deposited in the NCBI database under accession numbers SAMN50279182–SAMN50279188 and PX308679–PX308688.

## SUPPLEMENTAL MATERIAL

Tables S1 to S3 spectrum.02014-25-s0001.docx Table S1: Demographic Characteristics of the Study Population. Table S2: Geographic Setting. Table S3: The minimum inhibitory concentrations of tested antimicrobial agents against NDM-CRE isolates.

ASM does not own the copyrights to Supplemental Material that may be linked to, or accessed through, an article. The authors have granted ASM a non-exclusive, world-wide license to publish the Supplemental Material files. Please contact the corresponding author directly for reuse.

## References

[1] Zhang X-F, Peng L, Ke Y-F, Zhao D-D, Yu G-Q, Zhou Y, Li X, Weng X-B. 2023. Emergence of a clinical isolate of E. coil ST297 co-carrying bla_NDM-13_ and mcr-1.1 in China. J Infect Public Health 16:1813–1820. doi:10.1016/j.jiph.2023.09.007 37741016

[2] Taft DH, Liu JX, Maldonado-Gomez MX, Akre S, Huda MN, Ahmad SM, Stephensen CB, Mills DA. 2018. Bifidobacterial dominance of the gut in early life and acquisition of antimicrobial resistance. mSphere 3:1–24. doi:10.1128/mSphere.00441-18 PMC6158511 30258040

[3] Wang Q-J, Sun J, Li J, Ding Y-F, Li X-P, Lin J-X, Hassan B, Feng Y-J. 2017. Expanding landscapes of the diversified mcr-1-bearing plasmid reservoirs. Microbiome 5:1–9. doi:10.1186/s40168-017-0288-0 28683827 PMC5500976

[4] Luo Q, Wang Y, Fu H, Yu X, Zheng B, Chen Y, Berglund B, Xiao Y. 2020. Serotype Is associated with high rate of colistin resistance among clinical isolates of Salmonella. Front Microbiol 11:592146. doi:10.3389/fmicb.2020.592146 33391208 PMC7775366

[5] Wang S, Xie H-J, Chen Y-Z, Liu L, Fang M, Sun D-P, Xu L-C, Bi Z-W, Sun G-X, Li Y, Yu X-L, Zhang H-N, Kou Z-Q, Zheng B-W. 2022. Intestinal colonization with ESBL-producing Klebsiella pneumoniae in healthy rural villager: a genomic surveillance study in China, 2015-2017. Front Public Health 10:1–12. doi:10.3389/fpubh.2022.1017050 PMC9798286 36589964

[6] Song J, Oh S-S, Kim J, Park S, Shin J. 2020. Clinically relevant extended-spectrum β-lactamase–producing Escherichia coli isolates from food animals in South Korea. Front Microbiol 11:604. doi:10.3389/fmicb.2020.00604 32390965 PMC7188773

[7] Ma J, Xu R, Li W, Liu M, Ding X. 2024. Whole-genome sequencing of clinical isolates of Citrobacter europaeus in China carrying bla_OXA−48_ and bla_NDM−1_. Ann Clin Microbiol Antimicrob 23:1–9. doi:10.1186/s12941-024-00699-y 38685062 PMC11059591

[8] Yao X, Doi Y, Zeng L, Lv L-C, Liu J-H. 2016. Carbapenem-resistant and colistin-resistant Escherichia coli co-producing NDM-9 and MCR-1. Lancet Infect Dis 16:288–289. doi:10.1016/S1473-3099(16)00057-8 26842777

[9] Passet V, Brisse S. 2015. Association of tellurite resistance with hypervirulent clonal groups of Klebsiella pneumoniae. J Clin Microbiol 53:1380–1382. doi:10.1128/JCM.03053-14 25631812 PMC4365200

[10] Goessweiner-Mohr N, Arends K, Keller W, Grohmann E. 2014. Conjugation in gram-positive bacteria. Microbiol Spectr 2:PLAS-0004-2013. doi:10.1128/microbiolspec.PLAS-0004-2013 26104193

