# Supplementary material for: Emergence of coexisting blaNDM and mcr-1 genes in Escherichia coli isolates from the guts of healthy individuals
Source: Microbiol Spectr. 2025 Dec 23;14(2):e02014-25. doi: 10.1128/spectrum.02014-25 (PMC12889101; doi:10.1128/spectrum.02014-25)
Supplement: Tables S1 to S3 — Table S1: Demographic Characteristics of the Study Population. Table S2: Geographic Setting. Table S3: The minimum inhibitory concentrations of tested antimicrobial agents against NDM-CRE isolates. [file spectrum.02014-25-s0001.docx]

**Table S1.** **Demographic Characteristics of the Study Population (n=628)**

| Characteristic | Group | N | % / Mean±SD |
| --- | --- | --- | --- |
| Age (years) | ＞60 | 199 | 75.83±5.214 |
|  | 60-69 | 276 | 64.21±2.887 |
|  | ≥70 | 153 | 75.83±5.214 |
| Characteristic | Group | N | % |
| Gender | Male | 501 | 79.78% |
|  | Female | 127 | 20.22% |
| Education Level | Illiterate/Semi-literate | 207 | 33.0% |
|  | Primary School | 257 | 40.9% |
|  | Junior High+ | 164 | 26.1% |
| Farming Status | Non-farmers | 523 | 83.3% |
|  | Farmers | 105 | 16.7% |
| Animal Contact | Rare | 496 | 79.0% |
|  | Frequent | 132 | 21.0% |

The population of this study was predominantly male (≈80%), middle-aged to elderly (60–69 years as the largest group), with low education levels (≈74% had primary school or below). Most were non-farmers (83%) and had limited animal contact (79%).

**Table S2. Geographic Setting**

| Villages | Longitude | Dimension |
| --- | --- | --- |
| A | 119.483529 | 35.873418 |
| B | 119.48833 | 35.91174 |
| C | 119.484198 | 35.836701 |
| D | 119.474959 | 35.850233 |
| E | 119.513312 | 35.873527 |
| F | 119.509602 | 35.877922 |
| G | 119.523099 | 35.902093 |
| H | 119.513626 | 35.921474 |
| I | 119.431961 | 35.894385 |
| J | 119.41793 | 35.93449 |
| K | 119.465657 | 35.928262 |
| L | 119.480254 | 35.924659 |

**Table S3.** The minimum inhibitory concentrations of tested antimicrobial agents against NDM-CRE isolates. Resistance (R) is indicated in blue, susceptibility (S) is indicated in yellow, and intermediary (I) is indicated in pale yellow. CHL, chloramphenicol; SXT, trimethoprim-sulfamethoxazole; CT, colistin; ETP, ertapenem; MEM, meropenem; CTX, cefotaxime; CAZ, ceftazidime; CZA, ceftazidime-avibactam; TET, tetracycline; TIG, tigecycline; CIP, ciproffoxacin; NAL, naphthyridine acid; AZM, azithromycin; AMI, amikacin; STR, streptomycin; AMP, ampicillin; AMS, ampicillin-sulbactam.

| Number | MIC | | | | | | | | | | | | | | | | |
| --- | --- | --- | --- | --- | --- | --- | --- | --- | --- | --- | --- | --- | --- | --- | --- | --- | --- |
|  | CHL | SXT | CT | ETP | MEM | CTX | CAZ | CZA | TET | TIG | CIP | NAL | AZM | AMI | STR | AMP | AMS |
| EC0141 | ＞32 | ＞8 | 8 | ＞8 | ＞2 | ＞16 | ＞16 | ＞8 | ＞16 | ≤0.25 | 1 | 16 | 4 | ≤4 | 32 | ＞32 | ＞32 |
| EC0311 | 32 | ＞8 | ＞8 | 4 | ＞2 | ＞16 | ＞16 | ＞8 | ＞16 | 0.5 | 0.5 | 8 | 8 | ≤4 | 32 | ＞32 | ＞32 |
| EC0131 | ＞32 | ＞8 | 8 | 8 | ＞2 | ＞16 | ＞16 | ＞8 | ＞16 | 0.5 | ＞2 | ＞32 | 64 | ≤4 | 16 | ＞32 | ＞32 |
| EC0521 | 8 | ≤0.5 | 8 | ＞8 | ＞2 | ＞16 | ＞16 | ＞8 | ＞16 | 0.5 | ＞2 | ＞32 | 8 | ≤4 | 8 | ＞32 | ＞32 |
| EC0571 | ＞32 | ＞8 | 2 | ＞8 | ＞2 | ＞16 | ＞16 | ＞8 | ＞16 | 0.5 | ＞2 | ＞32 | 8 | ＞64 | 32 | ＞32 | ＞32 |
| EC0251 | ＞32 | ＞8 | 8 | 4 | ＞2 | ＞16 | ＞16 | ＞8 | ＞16 | 0.5 | 0.5 | 8 | 64 | ≤4 | 32 | ＞32 | ＞32 |
| EC0551 | ≤4 | ≤0.5 | 8 | ＞8 | ＞2 | ＞16 | ＞16 | ＞8 | ＞16 | ≤0.25 | 0.25 | ＞32 | 16 | ≤4 | 8 | ＞32 | ＞32 |
